# Making crystals with a purpose; a journey in crystal engineering at the University of Bologna

**DOI:** 10.1107/S2052252517005917

**Published:** 2017-06-13

**Authors:** Dario Braga, Fabrizia Grepioni, Lucia Maini, Simone d’Agostino

**Affiliations:** a Dipartimento di Chimica ‘G. Ciamician’, Università di Bologna, Via F. Selmi 2, Bologna 40126, Italy

**Keywords:** cocrystals, solid solutions, polymorphism, luminescence, chirality

## Abstract

The conceptual relationship between crystal reactivity, stability and meta­stability, solubility and morphology on the one hand and shape, charge distribution, chirality and distribution of functional groups over the molecular surfaces on the other hand is discussed, *via* a number of examples coming from three decades of research in the field of crystal engineering at the University of Bologna. The bottom-up preparation of mixed crystals, co-crystals and photoreactive materials starting from molecular building blocks across the borders of organic, organometallic and metalorganic chemistry is recounted.

## Abstract 2

The bottom-up preparation of mixed crystals, co-crystals and photoreactive materials starting from molecular building blocks across the borders of organic, organometallic and metal–organic chemistry is described.

## 1. Introduction   

Crystal engineering is, in essence, the attempt of the chemist to gain control on the assembly of molecular/ionic building blocks in the solid state *via* non-covalent interactions, forcing the result of a crystallization process towards planned structural and physical properties – an extremely ambitious goal.

Almost 60 years ago Richard P. Feynman, during his famous talk: ‘There’s Plenty of Room at the Bottom’, said ‘I can hardly doubt that when we have some control of the arrangement of things on a small scale we will get an enormously greater range of possible properties that substances can have, and of different things that we can do’ (Toumney, 2009 ▸).

Since most useful/utilizable materials are solids, and most solids are crystalline, the strive for the ‘controlled arrangement of things on a small scale’ is essentially an effort to design and construct crystals from preformed molecular building blocks. Scientists have always dreamt of being able to obtain materials with desired properties starting from a knowledge of the properties of the molecular/ionic components of choice and of their spatial distribution and intermolecular interactions in the solid. Crystal engineering is essentially making crystals with a purpose. What this purpose could be depends on the motivations of the experimentalist and could be utilitarian, esthetical or driven by curiosity, or a combination of these (Desiraju, 1989 ▸; Desiraju, 1995 ▸; Braga *et al.*, 1999 ▸). The number and type of useful crystalline materials that can be, at least in principle, obtained on the basis of the crystal engineering paradigm is limited only by the imagination of the ‘crystal maker’, and the applications span from photoelectronics to non-linear optics, from chiral materials, pharmaceuticals, agrochemicals, to nutraceuticals and cosmetics, let alone ‘zeolitic like’ materials to filter, trap, activate molecules on a nano scale *etc*.

However, depending on the category of materials one is aiming to obtain, the construction criteria are different for molecular crystals and coordination networks. Molecular crystals are mainly built up *via* the self-assembly of molecules and/or molecular ions capable of intermolecular bonding, while coordination networks are constructed using metal coordination topology and multidentate ligand linkers capable of coordination bonds.

In both cases, the crystal making process implies the existence of a project and of a design strategy (Braga & Grepioni, 2006 ▸; Zhang & Zaworotko, 2014 ▸). This is also the basic difference between crystal engineering and crystallography. While crystallography focuses on the characterization of a solid, crystal engineering focuses on its synthesis, and requires the definition of a target material or target property and of a synthetic strategy to reach such targets.

As a matter of fact, crystal engineering would have gone nowhere without the knowledge and tools of crystallography, but it is also true that crystal engineering has provided appealing alternatives to the work of crystallographers. Many structural chemists have found in crystal engineering a good motivation to abandon the (often frustrating) ancillary role of service crystallography to start making their own compounds. On the other hand, many synthetic chemists have found new inspiration and new ideas for their synthetic skill and moved from the preparation of molecules to that of solid supramolecular assemblies of higher complexity (Braga, 2017 ▸).

A crystal engineering project implies the ability to master a variety of energetically different supramolecular bonding interactions. These range from van der Waals to hydrogen bonding (Steiner, 2002 ▸; Jeffrey, 1997 ▸) and halogen bonding (Metrangolo *et al.*, 2008 ▸; Priimagi *et al.*, 2013 ▸; Cavallo *et al.*, 2016 ▸) from ligand-to-metal coordination bonds (Mensforth *et al.*, 2013 ▸; Batten & Champness, 2017 ▸) to ionic interactions, which also differ in terms of directionality, transferability and relative strength.

However, irrespective of the nature of the principal interactions, it should always be kept in mind that a molecular crystal represents a compromise between several, often non-converging factors, which, in the case of close neighbors, can also be repulsive in nature (Gavezzotti, 2013*a* ▸,*b* ▸; Dunitz *et al.*, 2014 ▸). Repulsions are effective at very short distances and much dependent on the nature of the peripheral atoms, which determine the electrostatic potential hypersurface surrounding the molecule. In this way the bulk of the molecule provides attraction, while surface atoms determine recognition, optimum relative orientation and interlocking of molecules in the solid state. In general, a given supramolecular arrangement in the solid state can be seen as the result of the minimization of short-range repulsions, rather than the optimization of attractions. It is therefore important, when considering a molecular crystal, to focus on the relationship between molecular shape and nature of the peripheral atoms. This is particularly relevant in the case of structurally non rigid molecules.

Weak bonds, such as C—H⋯O, C—H⋯N or C—H⋯π, play a role as ancillary interactions, whose optimization often determines the fine tuning of the crystal packing, while self-assembly is controlled by the stronger and more directional interactions (Desiraju & Steiner, 1999 ▸; Braga & Grepioni, 2000 ▸). The recognition process will be controlled by the outer shape of the molecule and by the nature of the peripheral atoms. The formation of a stable dimolecular aggregate – as the initial step of a crystallization process – whether formed by the same molecule, *i.e.* *AA*, or by two different molecules/ions, *i.e.* *AB*, or *A* ^+/−^ *B* ^−/+^, might depend primarily on the complementarity of shape.

This concept was elegantly expressed by L. Pauling long ago: ‘…in order to achieve the maximum stability, the two molecules must have complementary surfaces, like die and coin, and also a complementary distribution of active groups. The case might occur in which the two complementary structures happened to be identical; however, in this case also the stability of the complex of two molecules would be due to their complementariness rather than their identity’ (Pauling & Delbrück, 1940 ▸).

Over the past three decades many researchers have concentrated their efforts on dissecting, partitioning and ranking intermolecular bonding essentially on the basis of pairwise interactions (van der Waals, hydrogen bonds, halogen bonds *etc.*). This essential ‘taxonomy’ practice has been instrumental to devise synthetic strategies and to organize transmission of information between scientists aiming to preparing new crystalline materials with new or improved properties. However, the assembly of molecules does not usually follow the deterministic engineering approach. This is because the structure of a molecular crystal is only a minimum, often not even the deepest, in the thermodynamic landscape of a crystal. This understanding is fundamental if one plans to exploit the different types of interactions in the construction processes and also helps understand why this construction process often fails to reach the desired target. In many cases only metastable minima are easily reached, while stable minima can be elusive and remain undiscovered.

The actual occurrence of molecular crystal polymorphism (Bernstein, 2002 ▸; Brittain, 1999 ▸; Cruz-Cabeza *et al.*, 2015 ▸; Bučar *et al.*, 2015 ▸; Hilfiker, 2006 ▸; Cruz-Cabeza & Bernstein, 2014 ▸), for example, is still an unpredictable phenomenon, mainly because one can establish the geometry of a vast series of aggregates with a great degree of confidence but is unable to predict how the plethora of weak interactions (both attractive and repulsive) arising from next neighbors in molecular arrays will be optimized. And this in spite of the substantial progress made in the development of computational crystal structure prediction tools (Price, 2014 ▸).

But crystal engineering is also fun and scientifically rewarding. In the following, examples coming from the work of our group at the University of Bologna will be used to address some relevant aspects and criticisms of crystal engineering with organic and organometallic molecules.

## 2. Shape mimicry and the difference between organic and organometallic molecules   

**Figure 1:**
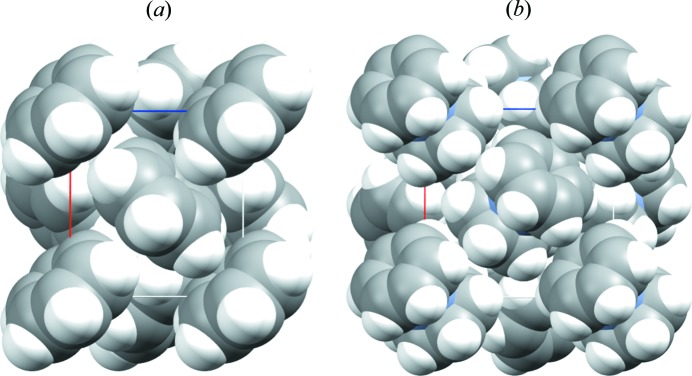
Space-filling comparison of the packing in crystalline benzene (CSD refcode BENZEN) and bis­benzene chromium (CSD refcode BBENCR03).

Our first attempt to make crystals on purpose was the consequence of a stimulating question asked by Margaret (Peggy) Etter at the Italo-Israeli meeting in Tel Aviv in 1992. By looking at the strong analogy in packing between benzene and (bis­)benzene chromium (see Fig. 1 ▸) we were showing, Professor Etter wondered if it was possible to predict the formation of an analogous (bis­)benzene chromium adduct of her benzene–cyclo­hexanedione co-crystal reported in 1986 (Etter *et al.*, 1986 ▸).

**Figure 2:**
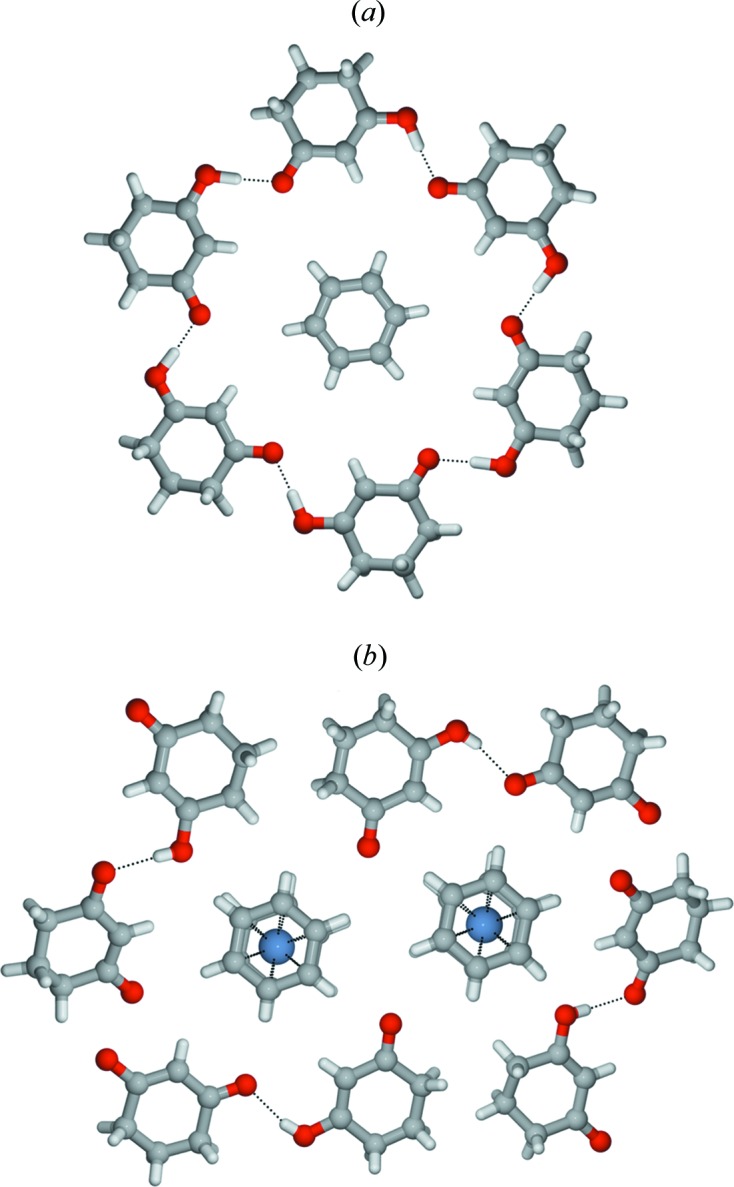
Comparison between Etter’s cyclamer and the encapsulation of bis­benzene chromium cations by neutral and anionic cyclo­hexane­diones.

The answer came a few years later and was somewhat unexpected (Fig. 2 ▸). Indeed, bis­benzene chromium could be encapsulated within a ‘belt’ of organic molecules similar to Etter’s cyclamer, but with the notable difference that the reaction had led to oxidation of bis­benzene chromium to the paramagnetic [Cr(η^6^-C_6_H_6_)_2_]^+^ cation and to deprotonation of one molecule of cyclo­hexane­dione (Braga *et al.*, 1995 ▸).

From this early experiment we learned not only that shape mimicry could be successfully used to envisage organometallic analogues of organic aggregates, but also that variable oxidation states of metal centers in organometallic compounds could play a crucial role in establishing the nature and electronic features, hence the potential applications as materials, of the products.

It also became clear that the combined use of ionic charges (*viz.* Coulombic interactions) and hydrogen-bonding interactions could be used to obtain electrostatically reinforced hydrogen bonds (charge-assisted hydrogen bonds) and properties that are in between those of molecular (neutral) crystals and those of molecular salts. The favorable location of ionic charges enhances both proton acidity and acceptor basicity in the solid state. Charge assistance to the hydrogen bond is the enhancement of donor and acceptor systems polarity, and it can be achieved by utilizing cationic donors and anionic acceptors instead of neutral systems, *i.e.* *X*—H(+)⋯*Y*(−) rather than *X*—H⋯*Y*.

**Figure 3:**
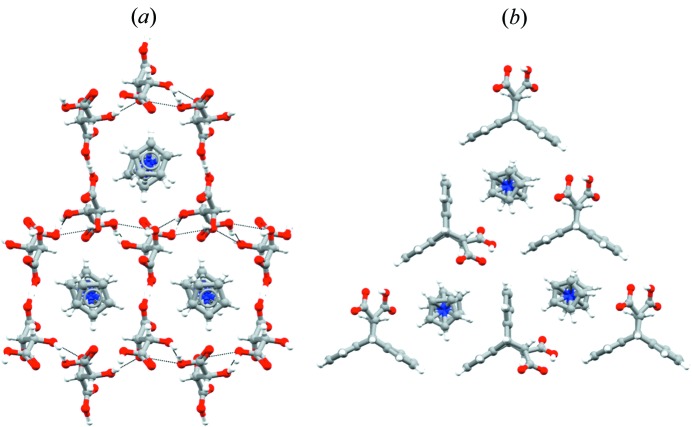
Representation of honeycomb frameworks formed by anions templated around cobaticinium cations in (*a*) [Co(η^5^-C_5_H_5_)_2_][(d,l-taH)·(d,l-taH_2_)] (d,l-taH_2_ = d,l-tartaric acid, CSD refcode NIGQID01) and (*b*) in [(η^5^-C_5_H_5_)_2_Co][*trans*-deccaH] (*trans*-DeccaH_2_ = *trans*-9,10-di­hydro-9,10-ethano­anthracene-11,12-di­carb­oxy­lic acid, CSD refcode BEKKAF).

A third relevant design element was the use of cylindrical templates, *viz.* the use of building blocks with cylindrical shapes, such as those of the organometallic sandwich compounds [Cr(η^6^-C_6_H_6_)_2_]^+^, Fe(η^5^-C_5_H_5_)_2_, [Co(η^5^-C_5_Me_5_)_2_]^+^ *etc.*, which could be used not only to synthesize and characterize a whole family of hybrid organic organometallic crystals (Braga, Giaffreda *et al.*, 2006 ▸), but also to assemble in honeycomb fashion organic molecules without the assistance of hydrogen bonds (Braga, d’Agostino & Grepioni, 2012*b* ▸), see Fig. 3 ▸.

## 3. From mechanochemical acid–base reactions to co-crystals   

Co-crystals are undoubtedly at the forefront of crystal engineering research. However, crystallization from solvents, the conventional method to grow crystals, has severe limitations when applied to multicomponent systems, *e.g.* co-crystals. As a matter of fact, strong differences in the solubility of the chosen components in a given solvent is often an unsurmountable obstacle to the formation of multicomponent systems. Clearly, the least soluble component will tend to precipitate first, usually causing the co-crystallization to fail.

The case may be that the co-crystal nucleus is rapid enough to form, and stable enough to grow, before nucleation of the crystals of the least soluble compound takes place, and yet it is always hard to predict whether the association of different molecules will be preferred to the homo-molecular aggregation. We found that mechanochemical methods used by us and others in different areas of chemistry (Shan *et al.*, 2002 ▸; Kaupp, 2009 ▸; Do & Friščić, 2017 ▸) could provide a route to overcome this problem. Mechanical mixing of components, however, brings about another significant problem: namely the lack of single crystals for precise determination of the structure of the multicomponent material. This further difficulty can be overcome in either of two ways: by determining the structure of the polycrystalline material directly from powder (*see below*), or by growing, *via* seeding, single crystals of the product obtained mechanochemically (Cherukuvada & Nangia, 2014 ▸; Braga & Grepioni, 2005 ▸).

**Figure 4:**
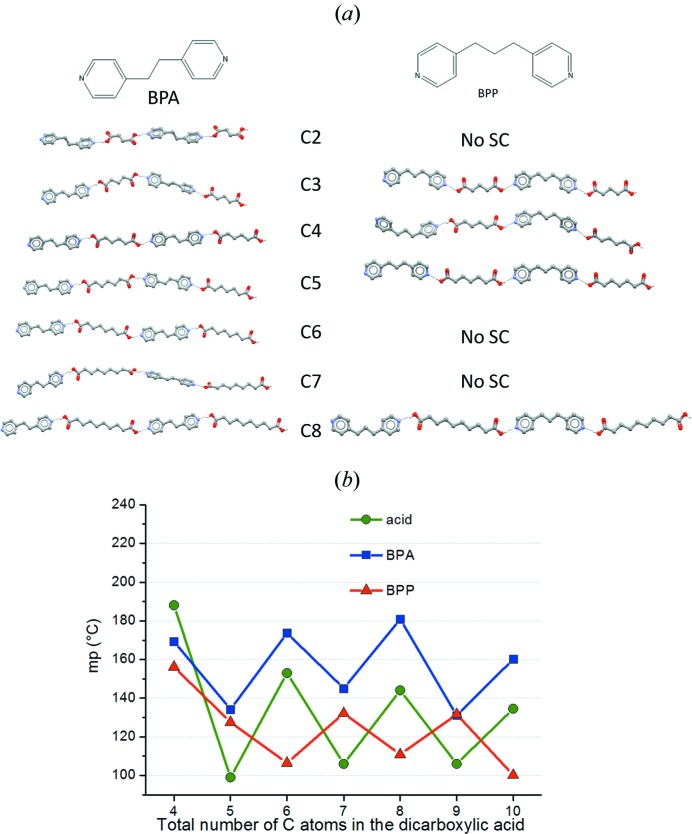
(*a*) Hydrogen-bonded chains of base and acid units in co-crystals of bis­(4-pyridyl)­ethene (BPE), and 1,2-bis­(4-pyridyl)­propane (BPP) with di­carb­oxy­lic acids with the total number (C_*n*_) of carbon atoms in the diacid molecule ranging between 4 and 10 (hence the adducts are identified as BPY·C_*n*_, BPA·C_*n*_, BPE·C_*n*_, and BPP·C_*n*_). (*b*) Melting point alternation found the even–odd alternating pattern associated with the sequence glutaric, adipic, pimelic, suberic, azelaic and sebacic acid was reversed in co-crystals BPP.

One of the early experiments of mechanochemical preparation of co-crystals along these lines was carried out in 2003, with the preparation of co-crystals of DABCO [DABCO = 1,4-di­aza­bicyclo­(2.2.2)octane] with di­carb­oxy­lic acids of variable aliphatic chain length (Braga *et al.*, 2003 ▸). It was observed that, depending on the even/odd number of carbon atoms in the di­carb­oxy­lic acid chain, the products showed melting point alternation analogous in trend to that observed for the pure acids, the co-crystals with odd aliphatic chain having a lower melting point than those with an even number of C atoms in the chain. Interestingly, in a follow up study, we showed that the sequence could be inverted by using a base with an odd number of atoms, showing that it was the total even/odd number of atoms in the unit reproduced periodically in the crystal that was associated with the melting point trend (see Fig. 4 ▸) (Braga *et al.*, 2010 ▸).

**Figure 5:**
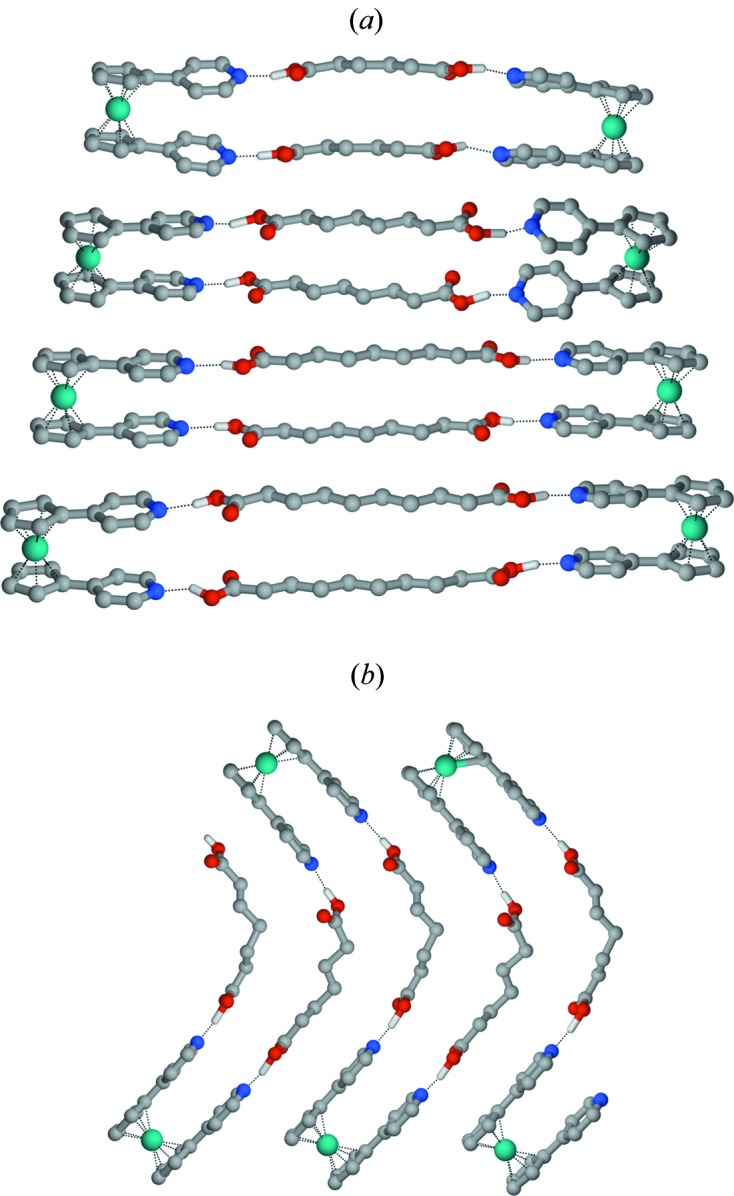
(*a*) The supramolecular structures of the macrocycles {[Fe(C_5_H_4_–C_5_H_4_N)_2_]·[HOOC(CH_2_)_*n*_COOH]}_2_ [*n* = 4 (*a*), 6 (*b*), 7 (*c*), 8 (*d*)]; (*b*) the zigzag chain found when *n* = 5.

Mechanical mixing opened up the door to a number of experiments with organic, inorganic and organometallic systems, and to the exploration of alternative ways to obtain crystal polymorphs. Another example is provided by the co-crystals obtained by reacting ferrocene bi­pyridine with carboxilic acids (see Fig. 5 ▸) (Braga *et al.*, 2008 ▸).

In the course of these studies we also learned that the outcome of the mechanochemical preparation was very much dependent on the wet or dry conditions of the reactants. The use of a small quantity of solvent (*kneading*, also called *solvent drop grinding* and later *liquid assisted grinding*) entered the synthetic strategy for the preparation of co-crystals (Braga *et al.*, 2013 ▸; James *et al.*, 2012 ▸).

## 4. From mechanochemical reactions to luminescent coordination polymers   

**Figure 6:**
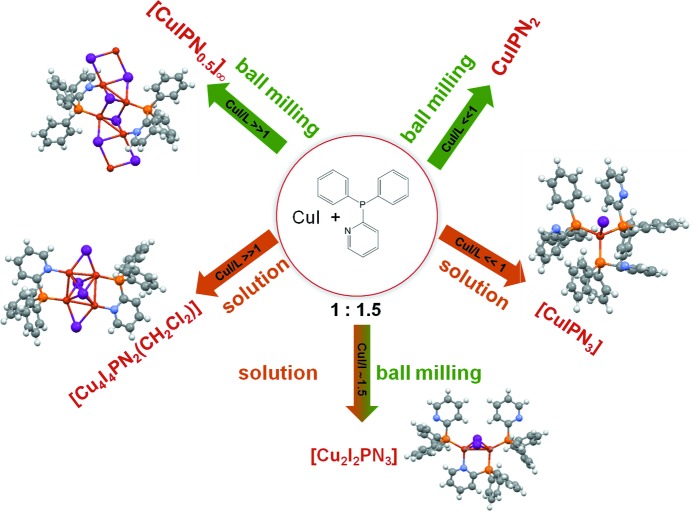
Comparison of solid-state and solution synthesis for the reaction of CuI with di­phenyl-2-pyridyl phosphine (PN), which yields five different crystal forms, depending on stoichiometry ratio and synthetic procedure.

Mechanochemical reactions offer a valid alternative to solution synthesis also for the preparation of coordination compounds and coordination polymers (Braga, Curzi *et al.*, 2006 ▸). Solid state synthesis has been used to prepare coordination compounds based on CuI, thus overcoming the problem of the poor reactant solubility. Copper(I) halide aggregates constitute a large family of compounds studied mainly for their strong luminescence at ambient temperature, especially in the solid state (Wallesch *et al.*, 2014 ▸) and they are characterized by the remarkable structural diversity on the CuX core (Peng *et al.*, 2010 ▸). By varying the CuI/ligand stoichiometry ratio it is possible to attain some degree of control of the nuclearity of the Cu_*x*_I_*x*_ core. Moreover, reactions performed in the solid state can yield crystal forms hardly or even not obtainable in solution. A useful example is the reaction of CuI with di­phenyl-2-pyridyl phosphine (PN), which yields five different crystal forms depending on stoichiometry ratio and synthetic procedure, as can be seen in Fig. 6 ▸ (Maini *et al.*, 2014 ▸). The dimer [Cu_2_I_2_(PN)_3_] can be easily obtained by solution or ball milling. When the ligand is present in excess, compounds with lower nuclearity are obtained, such as [CuI(PN)_3_] from solution and [CuI(PN)_2_] by ball milling. When CuI is present in excess, syntheses in solution and in the solid state yield [Cu_4_I_4_(PN)_2_(CH_2_Cl_2_)] and the infinite double chain [CuI(PN)_0.5_]_∞_, respectively.

## 5. From IR pellets to ionic co-crystals   

**Figure 7:**
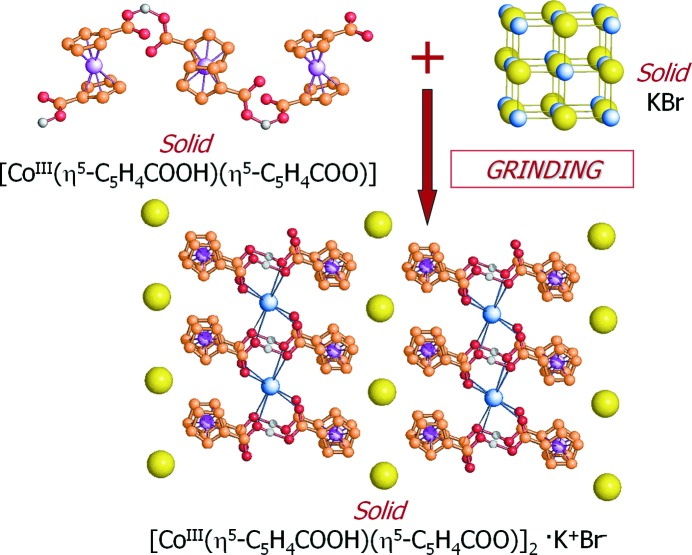
The accidental discovery of the formation of the supramolecular ‘crown ether’-like complexation of the cation in the KBr adduct [Co(C_5_H_4_COOH)(C_5_H_4_COO)]KBr.

**Figure 8:**
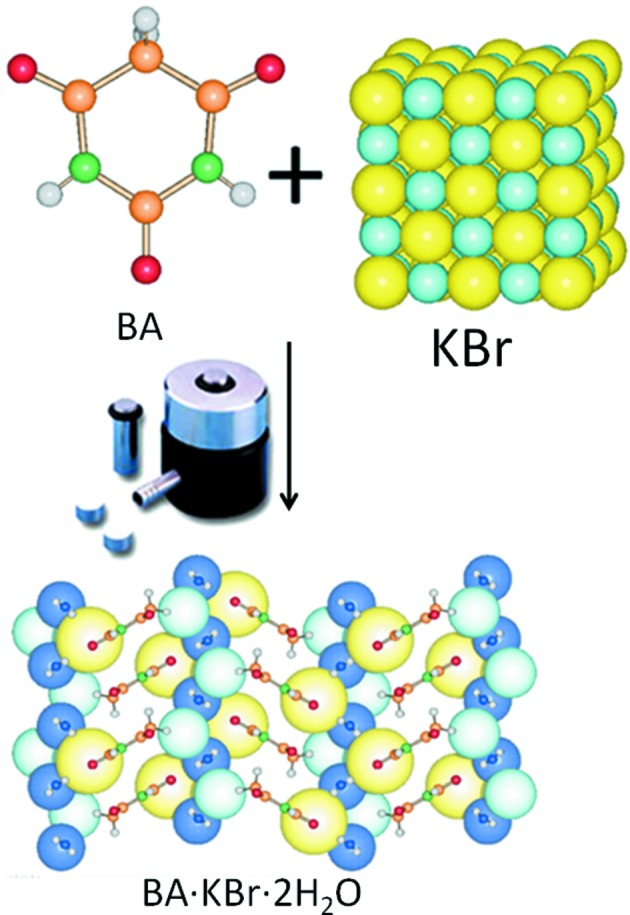
Mechanochemical preparation of the ionic co-crystal between barbituric acid and KBr.

The ‘mechanochemical’ awareness developed in the investigation of many solid–solid reactions (Braga & Grepioni, 2004 ▸) allowed us to understand the unexpected result observed in the course of the preparation of IR pellets of the organometallic zwitterion [Co(C_5_H_4_COOH)(C_5_H_4_COO)]. The appearance of unexpected bands could be rationalized envisaging the formation of a new compound between the zwitterion and KBr (Braga *et al.*, 2002 ▸). Fig. 7 ▸ summarizes the result of the ‘on purpose’ preparation of co-crystals of the cobalt(III) zwitterion [Co(C_5_H_4_COOH)(C_5_H_4_COO)] with KBr, which was then extended to a whole class of supramolecular complexes, whereby the alkali cations had been trapped in a sort of crown-ether fashion with the halogen ions on the outside of the organometallic scaffolds. Following these early discoveries, we learned that it was possible to produce ‘on purpose’ organic–inorganic ionic co-crystals (ICCs) between an organic molecule and an inorganic salt (see Fig. 8 ▸). If the organic molecule is an API or an API precursor, pharmaceutical ionic co-crystals are obtained (Braga *et al.*, 2010 ▸; Braga, Grepioni *et al.*, 2012 ▸). In these cases the combination between an ionic crystal and an API can alter the physicochemical properties of the solid in a significant manner.

In the pharmaceutical field the design of co-crystals where the co-former is an inorganic salt is still an almost unexplored subject (Oertling, 2016 ▸; Duggirala *et al.*, 2016 ▸). The stability of the ionic co-crystals depends on the interactions established by the organic moiety with cations and anions, respectively, which resemble the interactions that solvent molecules establish with ions in solution.

**Figure 9:**
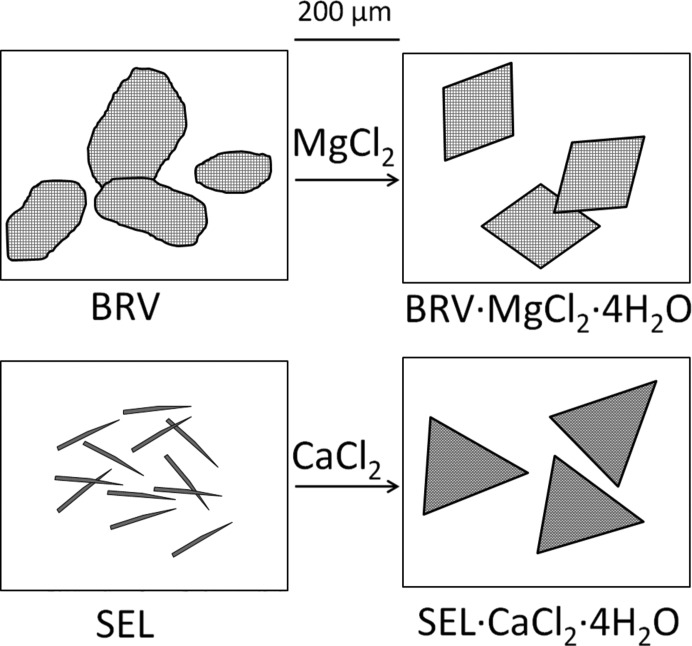
Comparison between crystal morphology in crystalline BRV_2_·MgCl_2_·4H_2_O and SEL·CaCl_2_·2H_2_O.

Another example of pharmaceutical ICCs is provided by the co-crystals of brivaracetam (BRV) and seletracetam (SEL) with lithium salts, namely BRV_2_·LiBr, SEL_2_·LiBr and SEL·LiCl·2H_2_O, synthesized by grinding and/or solvent evaporation. These lithium-racetams ICCs constitute an entire new class of co-crystals, which combine the medical properties of the racetams and those of the lithium salts (Grepioni *et al.*, 2014 ▸). Analogously, BRV and SEL were co-crystallized with the inorganic salts MgCl_2_ and CaCl_2_, yielding BRV_2_·MgCl_2_·4H_2_O, SEL_2_·MgCl_2_·4H_2_O, BRV·CaCl_2_·2H_2_O and SEL·CaCl_2_·2H_2_O. Properties like filtering and flowability, as well as melting point, hygroscopicity, crystal morphology can be modified (see examples in Fig. 9 ▸).

## 6. From chiral co-crystal metathesis to ionic selection   

**Figure 10:**
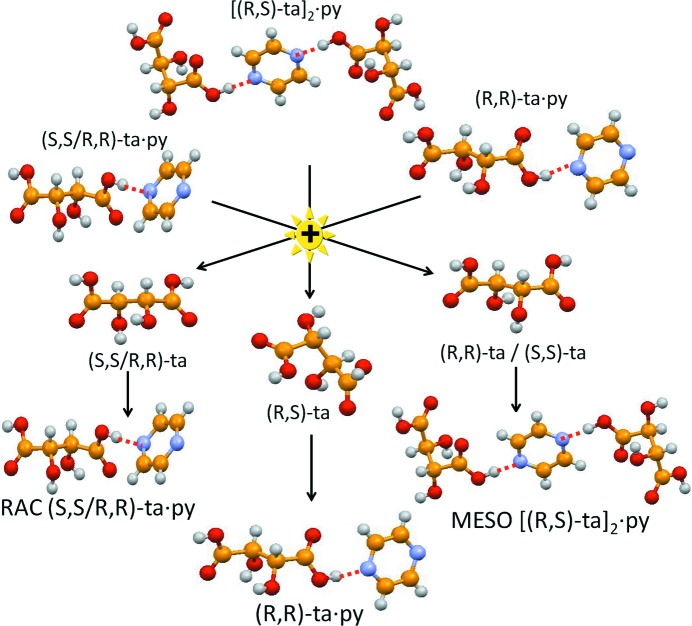
Relationship between the various co-crystals obtained from *meso*-, *rac*-, and enantiopure tartaric acid and pyrazine.

Solid-solid reactions with co-crystals or between co-crystals can be used as an alternative to conventional reactions in solution to produce co-crystals as well as to interconvert co-crystals. In the case of the co-crystals formed by the different isomers of tartaric acid and pyrazine, it was possible to establish an empirical ranking of crystal stability by ‘reacting’ preformed co-crystals with *meso*-, enantiopure and racemic tartaric acid. This sort of supramolecular metathesis yielded the scale of relative crystal stability [(*R*,*S*)-ta]_2_·py > (*S*,*S*/*R*,*R*)-ta·py > [(*S*,*S*/*R*,*R*)-ta]_2_·py > (*R*,*R*)-ta·py or (*S*,*S*)-ta·py (Braga *et al.*, 2011 ▸) (see Fig. 10 ▸). Structural effects of chiral *versus* racemic tartaric acid in the synthesis of adducts with di­amines have also been investigated by other scientists (Aakeroy *et al.*, 1992 ▸; Farrell *et al.*, 2002 ▸). Co-crystallization has also been used in chiral resolution (George *et al.*, 2016 ▸; Springuel & Leyssens, 2012 ▸; Springuel *et al.*, 2014 ▸).

**Figure 11:**
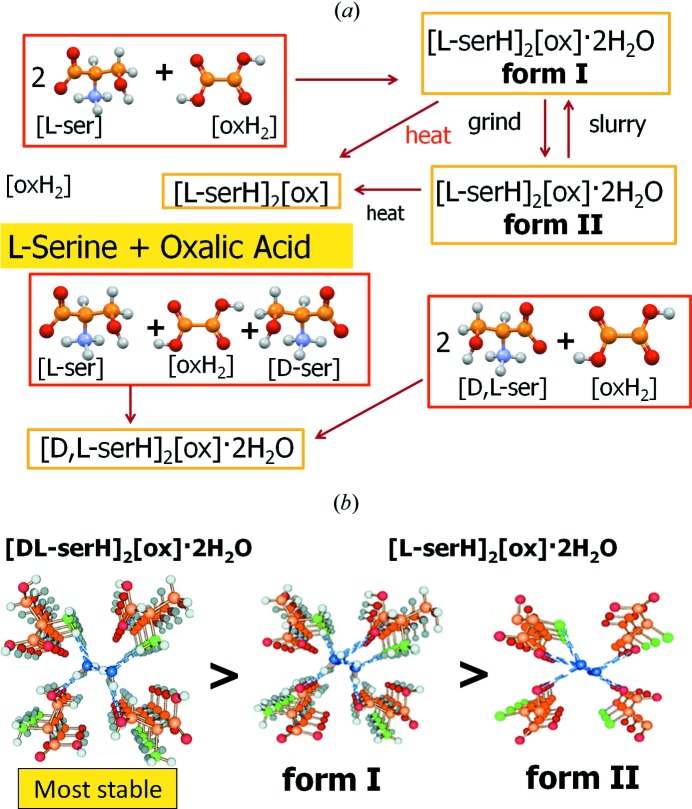
(*a*) Relationship between the various crystal forms obtained by reacting l- and dl-serine with oxalic acid; (*b*) note the strong resemblance between crystals of dl and l-serine oxalate hydrates.

In yet another set of experiments with enantiopure and racemic molecules, l-serine and dl-serine were treated with oxalic acid under different experimental conditions, *e.g.* crystallization from solution and slurry, kneading and dry mixing, yielding two crystal forms of the molecular salts [l-serH]_2_[ox]·2H_2_O as well as salts of the formula [l-serH][Hox] and [dl-serH]_2_[ox]·2H_2_O, which show an intriguing structural relationship and a strong resemblance between the enantiopure and the racemic materials (Braga *et al.*, 2013 ▸) (see Fig. 11 ▸).

**Figure 12:**
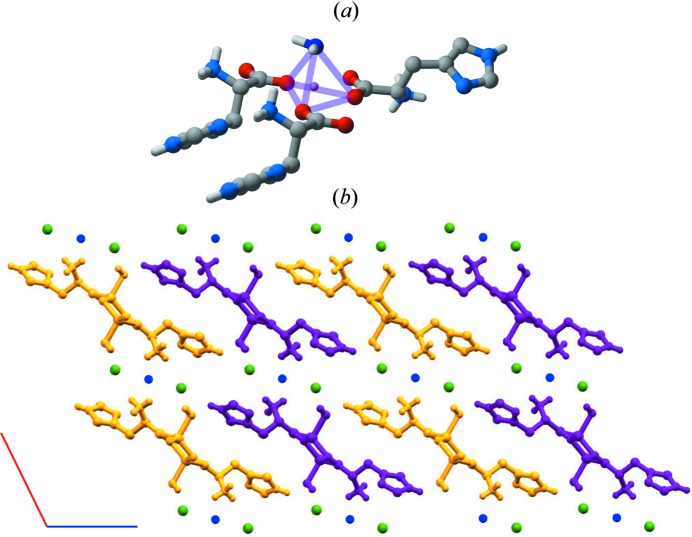
(*a*) Coordination around the lithium ion, and (*b*) view of the crystal packing along the *b*-axis, showing alternate, enantiopure chains, here indicated in violet and orange, containing only l- or d-histidine ligands (O_water_ in blue, Cl^−^ in green).

More recently, mechanochemical methods of preparation have been used to investigate the formation of ICCs of l- and dl-histidine with lithium halides Li*X*, *X* = Cl, Br and I (Braga *et al.*, 2016 ▸). The overall picture that emerged from this study was intriguing, since conglomerate formation of the l and d separate enantiomers was observed in the case of LiI, with spontaneous chiral resolution and formation of enantiopure crystals l-His·LiI·1.5H_2_O and d-His·LiI·1.5H_2_O. In the cases of LiCl and LiBr, on the other hand, the racemic crystals dl-His·LiCl/Br·1.5H_2_O presented a chiral preference within the crystal, to the extent that the product could also be described as a kind of ‘co-crystal’ formed by enantiopure l-His·LiCl/Br·H_2_O and d-His·LiCl/Br·H_2_O joined by water bridges, as shown in Fig. 12 ▸.

## 7. From isomorphous crystals to solid solutions   

When molecular size and shape are very similar, it is possible that the components become miscible in a range of compositions, as in the case of alloys.

The importance of non-stoichiometric molecular mixed crystals as novel functional materials has been recently emphasized in a study of mixed crystals of acridine and phenazine prepared mechanochemically (Schur *et al.*, 2015 ▸). This approach has been expanded to the preparation of ternary mixed crystals of anthracene, phenazine and acridine (Lusi *et al.*, 2015 ▸). Mixed crystals will certainly provide access to a broad variety of new properties, as it has been amply demonstrated in other cases of molecular alloying, also with organometallic molecules and salts (Braga *et al.*, 2001 ▸; Steed *et al.*, 2007 ▸; Nangia & Cherukavada, 2014 ▸).

**Figure 13:**
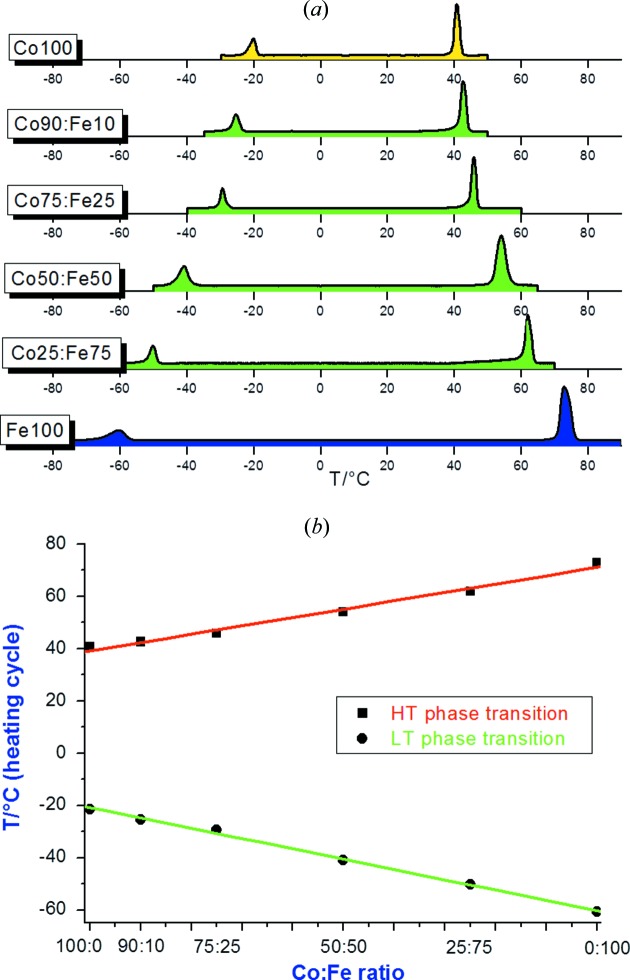
Representation of the thermal behavior of the mixed crystals [Co_*x*_Fe_1 − *x*_(η^5^-C_5_H_5_)_2_][PF_6_]. Note the linear, and divergent, change in transition temperature on increasing the iron percentage in the solid solution.

The two isomorphous crystalline complexes [*M*(η^5^-C_5_H_5_)_2_][PF_6_] (*M* = Co, Fe) form enantiotropic polymorphs that interconvert as a function of temperature, by undergoing two reversible solid-to-solid phase changes towards a low-temperature monoclinic phase and a high-temperature cubic phase, respectively (Braga *et al.*, 2001 ▸). The only difference between the salts of two metal complexes is in the transition temperatures, which occur at 213.1 and 347.1 K in the case of Fe, and at 251.8 and 313.9 K in the case of Co. The two salts are fully miscible in the whole range of composition, resulting in mixed salts of the formula [Co_*x*_Fe_1 − *x*_(η^5^-C_5_H_5_)_2_][PF_6_] (with 0 < *x* < 1). The phase transition behavior depends linearly on the composition, *i.e.* the temperatures at which the two solid-to-solid phase transitions occur can be selected by choosing the molar ratio in solution. Thus, the mixed-crystal [Co_*x*_Fe_1 − *x*_(η^5^-C_5_H_5_)_2_][PF_6_], though composed of molecular ions and soluble in water, possesses the features of an alloy of the *A*_*x*_ *B*_1 − *x*_ type (see Fig. 13 ▸).

**Figure 14:**
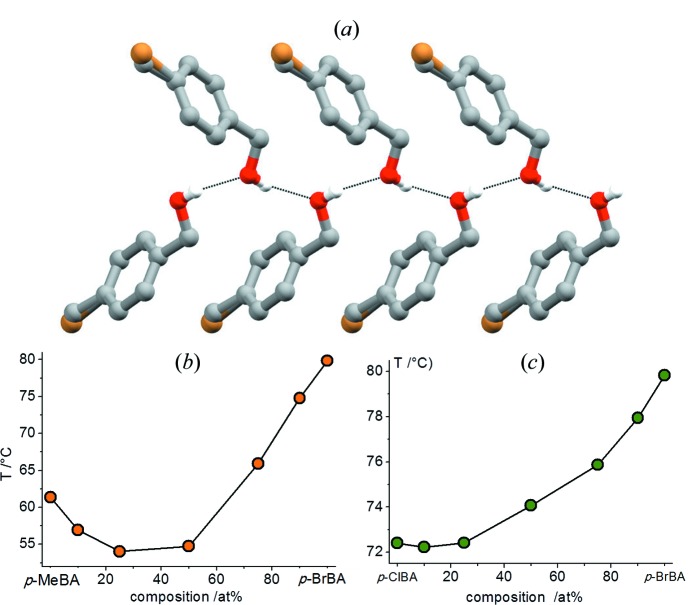
Crystal packing of the 50:50 MeBA/BrBA solid solution (projection in the *bc*-plane). Left: melting points *versus* atomic fraction of BrBA in MeBA_1 − *x*_BrBA_*x*_ solid solutions. Right: melting points of the ClBA_1 − *x*_BrBA_*x*_ solid solutions obtained by co-melting.

**Figure 15:**
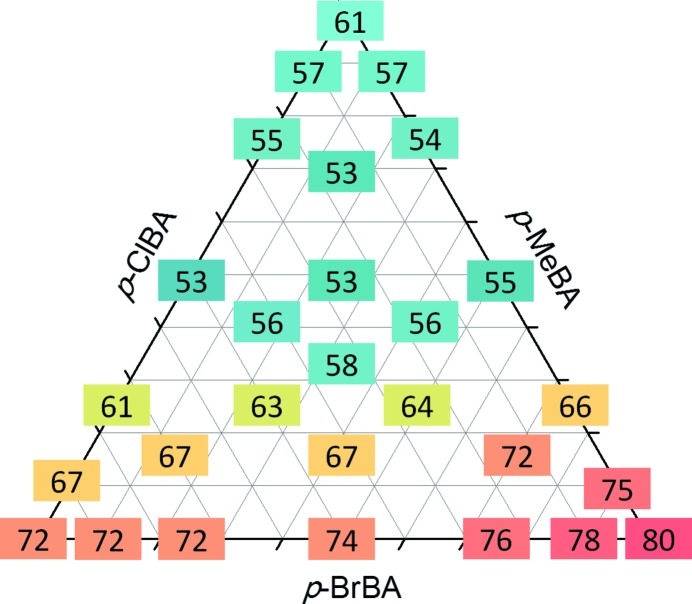
A ternary diagrams showing the melting points (°C, peak temperatures from DSC) for various compositions of the MeBA_1 − *x* − *y*_ClBA_*x*_BrBA_*y*_ solid solutions.

With this awareness, we have investigated other series of isomorphous crystals formed by quasi-isostructural molecules, such as *p*-chloro­benzyl alcohol (ClBA), *p*-bromo­benzyl alcohol (BrBA) and the quasi-isostructural compound *p*-methyl­benzyl alcohol (MeBA). The binary, MeBA_1 − *x*_BrBA_*x*_ and ClBA_1 − *x*_BrBA_*x*_, and ternary, MeBA_1 − *x* − *y*_ClBA_*x*_BrBA_*y*_, solid solutions were synthesized by co-melting the crystalline solids in various molar ratios (Romasanta *et al.*, 2017 ▸) resulting in solid solutions having melting points changing with composition, as shown in Fig. 14 ▸. It was also possible to obtain ternary isomorphous crystals with a ‘tunable’ melting point as a function of the composition (see Fig. 15 ▸).

In an analogous case of quasi-isostructurality, co-crystals of *ortho*-toluic and *ortho*-chloro benzoic acid in 50:50 composition were obtained, while no alloying was observed (Polito *et al.*, 2008 ▸).

**Figure 16:**
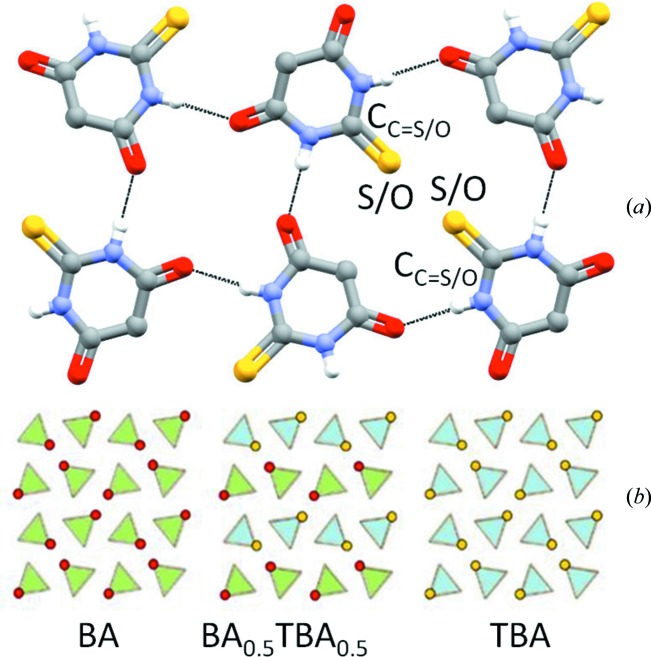
The co-crystal obtained by mixing barbituric acid and thio­barbituric acid in 1:1 composition.

Crystals of the quasi isostructural molecules barbituric and thio­barbituric acids are also isomorphous, and they can form solid solutions. Surprisingly, it was possible not only to prepare solid solutions of the general formula BA_*x*_TBA_1 − *x*_ (*x* < 0.8), but also to obtain a 1:1 co-crystal isomorphous with the parent molecular crystals as keto forms. The BA·TBA co-crystal melts at 265°C, *i.e. ca* 10 and 20 °C higher than homomolecular BA and TBA, respectively (Shemchuk *et al.*, 2016 ▸). While the BA_*x*_TBA_1 − *x*_ solid solutions with *x* > 0.5 are stable, those with *x* < 0.5 convert, upon time or temperature, to the BA_0.5_TBA_0.5_ co-crystal. The BA–TBA equimolar mixture generates a packing which appears to be more favored with respect to the parent homo-molecular crystalline materials (see Fig. 16 ▸).

## 8. From single-crystal-to-single-crystal transformations to solid solutions   

A special case of solid-state reactivity is observed when a reactant single crystal yields a product in the form of a single crystal, in response to a stimulus (thermal, luminous *etc.*) capable of triggering a physical or chemical transformation. This type of conversion is defined as single-crystal-to-single-crystal reaction or simply SCSC (Halasz, 2010 ▸; Aggarwal *et al.*, 2014 ▸). Solid state [2 + 2] photodimerizations (Braga, d’Agostino & Grepioni *et al.*, 2012*a* ▸; Biradha & Santra, 2013 ▸; Sinnwell & MacGillivray, 2016 ▸) represent a class of good candidates to realise such transformations especially when they proceed topotactically, *i.e.* with the least molecular motion (Ramamurthy & Sivaguru, 2016 ▸). When the reaction conditions are carefully balanced it is also possible to obtain partially reacted single crystals, *i.e.* solid solutions where the product molecules are ‘dissolved’ in the crystal lattice of the reactant (and *vice versa* after the 50% of conversion).

**Figure 17:**
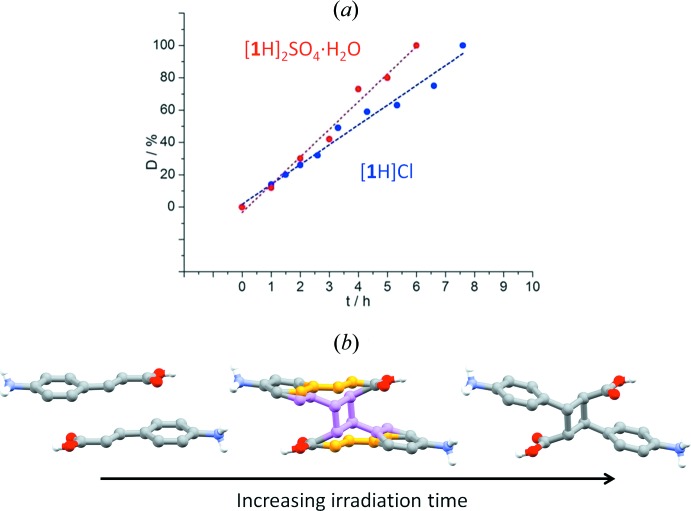
(*a*) Dependence of dimer content (D, %) on the irradiation time for the [**1**H]Cl and [**1**H]_2_SO_4_·H_2_O molecular salts, and (*b*) progressive photodimerization through the formation of solid solutions.

With this in mind we have prepared and investigated the [2 + 2] photoreactivity in a series of molecular salts with the general formula [**1**H]_*n*_ *A*·*x*H_2_O (**1** = 4-amino-cinnamic acid, *A* ^*n*−^ = NO_3_^−^, BF_4_^−^, PF_6_^−^, SO_4_2^−^, *x* = 0, 1). Amongst all, only the chloride and sulfate salts were found to undergo SCSC [2 + 2] photodimerization to generate the corresponding α-dimers containing crystals (d’Agostino *et al.*, 2016 ▸). Moreover, the conversions were followed stepwise by single-crystal X-ray diffraction: in both cases, upon irradiation, the presence in the crystals of the cyclo­butane ring was detected, and its percentage increased up to complete conversion, as shown in Fig. 17 ▸. The intermediate steps are representative of true solid solutions of monomer pairs and dimers for the whole range of compositions.

## 9. Conclusions   

Crystal engineering, namely the design, construction and exploitation of functional crystalline materials, has become the new frontier of solid-state chemistry. It has evolved from its supramolecular cradle to become not only a fully fledged academic field of research with dedicated courses, PhD theses, books, specific journals, meetings and research projects, but also an area of industrial interest (MOF production lines, patents on co-crystals *etc.*). This is because crystal engineering acts as a bridge between fundamental studies and applied research, providing the conceptual tools and the instruments to investigate elusive phenomena such as nucleation, solvate formation and crystal polymorphism, as well as to devise new materials for applications in a variety of sectors.

In this review we have connected some early findings to more recent results, obtained in our continuing exploration of the territory of crystal engineering. An exploration that has led us across traditional disciplinary barriers, demonstrating that crystal engineering has no border. We share the holistic view of Gautam Desiraju (Desiraju, 2013 ▸, 2017 ▸).

However, the full realisation of the crystal engineering paradigm – namely the exact, fully predictable, surprise-free engineering of a new crystal starting from the assembly of molecules, ions, and complexes – remains an elusive objective. The crystal maker may well predict with a high level of confidence whether a given functional group will link to another (the same or different) functional group in a predetermined way, or whether metal atoms would be involved in one-, two- or three-dimensional coordination with divergent ligands, but he/she is still unable to predict whether he/she will obtain the most thermodynamically stable form or only one of many kinetic alternatives.

Often the crystal maker is unable to predict even the exact chemical composition of a crystalline product, *e.g.* the possible formation of a solvate, precipitated for the first time from a solvent or mixture of solvents, or produced by grinding in a humid environment. Analogously, whether a co-crystal may be formed or not by reacting molecules in solution or in the solid state and whether the resulting crystalline product will be more stable than the separate homomolecular crystals can only be ascertained by trial-and-error experiments.

This lack of predictability, instead of weakening the motivation of the crystal makers, has strengthened the need for further experimentation and research. Not only this, the need to understand the conditions for guaranteeing persistence of crystal properties with time and an exact control on the physico-chemical properties when crystalline materials are administered to humans and animals (in the form of food, drugs, nutraceuticals, cosmetics *etc.*) has fuelled the systematic investigation of polymorphs and solvates, which, in turn, has provided the motivation for the birth of research-oriented companies. These laboratories, often academic spin offs, are able to intercept the demand of pharmaceutical, agrochemical and food industry for high level research in the solid-state area. Universities have also benefited from funding to academic research labs. One can have different opinions about the importance of industry–academy interactions, but the fact that nowadays the quest for crystal forms is such a crucial issue, and that many graduates trained in crystallography and solid-state chemistry techniques have a job in science because of this knowledge, is also a positive, largely unforeseeable result of the need to explore crystal landscapes. Another reason for ‘making crystals with a purpose’.
